# HARNESSING VOICE-ASSISTED TECHNOLOGY AND IN-HOME SENSORS TO MANAGE OLDER ADULT HEALTH: A USER PREFERENCE STUDY

**DOI:** 10.1093/geroni/igz038.3374

**Published:** 2019-11-08

**Authors:** Erin L Robinson, Geunhye Park, Shradha Shalini, Trevor Levins, Kari R Lane, Marjorie Skubic, Brianna Markway

**Affiliations:** 1 University of Missouri, School of Social Work, Columbia, Missouri, United States; 2 University of Missouri-Columbia, Columbia, Missouri, United States; 4 University of Missouri, College of Engineering, Columbia, Missouri, United States

## Abstract

In recent years voice-assisted technologies, such as the Amazon Echo Show and Google Home, have been harnessed to help older adults manage their health. However, little is known about the use of such technologies in combination with in-home sensor systems to help older adults age in place. Therefore, this research explored user preferences of older adults and a designated family member/friend in using voice-assisted technologies to retrieve in-home sensor-generated health information, such as fall risk and other early indicators of health changes. Seventeen dyad interviews were conducted with known pairs of older adults (Mean age=75; 56% female) and a family member/friend (Mean age=64; 89% female). Participants were given a description of the technology and its capabilities, and then were instructed to interact with each device using a prepared scenario. Participants asked each device health-related questions to elicit pre-programmed information for the respective scenarios and provided user experience feedback for each device. At the end of the interview, participants completed a speech recognition test for each device and a technology acceptance survey. Overall acceptance of the technology was high, and participants believed that using voice-assisted technologies to retrieve sensor-generated health information would be beneficial in managing their health or providing care to a family member/friend. However, advantages and disadvantages exist with each device and the Google Home generally performed better on the speech recognition test for each dyad pair. These findings provide valuable insight about older adults’ preferences (as well as family members’/friends) in using voice-assisted technologies to manage their health.

